# Preterm Birth and the Type of Birth and Their Impact on the Incidence of Overweight and Obesity in Children

**DOI:** 10.3390/ijerph191912042

**Published:** 2022-09-23

**Authors:** Joanna Baran, Aneta Weres, Rafał Baran, Ewelina Czenczek-Lewandowska, Justyna Leszczak, Justyna Wyszyńska

**Affiliations:** 1 Institute of Health Sciences, Medical College, University of Rzeszów, 35-310 Rzeszów, Poland; 2 Natural and Medical Center for Innovative Research, 35-310 Rzeszów, Poland; 3 SOLUTION-Statistical Analysis, 35-120 Rzeszów, Poland

**Keywords:** type of delivery, preterm birth, obesity

## Abstract

The purpose of the study was to evaluate the influence of the type of birth and preterm birth on the risk of overweight and obesity in the children studied. The study involved 749 children of pre-school and school age, between 4 and 15 years of age. Information about the type of delivery and the potential preterm birth came from the child’s health book and the mother’s pregnancy card. The authors assessed the body height and body weight of each child. The analysis showed that on average every six children were born before due date (before the end of 37 weeks of gestation) and slightly more than 40% of the children were born by cesarean section (CS). A statistical analysis was performed, including descriptive statistics and Spearman’s correlation, and to evaluate the differences in the analyzed groups, nonparametric tests and chi-square independence tests were used: the Mann-Whitney test, and the Kruskal-Wallis test due to the lack of a normalized distribution. The incidence of overweight and obesity was higher in 7–11-year-old boys born with CS vs. vaginal birth (VD) (*p* = 0.026). There were no statistically significant differences between BMI centile value and preterm birth. Cesarean section birth significantly increases the percentage of boys with overweight and obesity in early school age and may be associated with higher percentile values of children with BMI in this age group.

## 1. Introduction

The problem of excessive body mass extends its scale both among pregnant women and in the pediatric population from the early ages of birth. The incidence of obesity among infants and young children (aged 0 to 5 years) around the world has increased rapidly from 32 million worldwide in 1990 to 42 million in 2013, and further growth is expected to reach 70 million by 2025 [1]. By 2022, the prevalence of childhood obesity may outweigh the prevalence of moderate and severe underweight in children [2]. A similar trend is observed in the case of pregnant women with overweight or obesity, whose number is constantly growing, mostly in high-income and middle-income countries, including mainly India, China, Nigeria and the USA [3]. Both of these phenomena are interrelated, and obesity in pregnancy can have a negative impact on the health of both the mother and her child in the future [4].

Numerous studies indicate that increased maternal weight contributes to a number of complications during pregnancy (e.g., premature births, low Apgar scores at 5 min, low birth weight, miscarriages, poor neonatal condition, or the need to perform a cesarean section (CS)) [5,6,7]. Women who are also overweight during pregnancy are more likely to have babies that become obese or overweight [8]. Simultaneously, they are also more likely to have a CS birth, and a systematic review showed that, compared to women who had physiological births, they have a 30% higher chance of having children with excessive body mass [9]. It is suggested that, similar to excessive body weight of the mother during pregnancy, CS itself may also be associated with a higher risk of later being overweight and obesity in children, as confirmed by numerous data from the literature [10,11,12]. 

Premature birth (also known as preterm) birth is when a baby is born too early, before 37 weeks of pregnancy have been completed [13]. According to the latest global estimates, the number of premature births each year is greater than 15 million, and similar to the case of CS section, it can expose a child to many problems throughout life, including obesity [14]. Children could have short-term problems (with breathing, immune system, heart, brain, gastrointestinal system, temperature control, blood, or metabolism) and long-term complications (vision problems, hearing problems, cerebral palsy, behavioral and psychological problems, impaired learning, and chronic health issues). At the same time, the risk of premature birth is known to increase proportionally to the increasing maternal weight of the pregnant woman [15]. Approximately 2–8% of all premature babies develop small-for-gestational-age children (PTSGAs), but longitudinal information about their growth and its association with preterm gestational age children (PT-AGA) is sparse [16]. The mechanisms of early weight gain in children subjected to intrauterine growth restriction may lead to obesity in childhood and adolescence following rapid weight gain. To grow at a rate similar to that of babies born at full term, premature babies must be properly fed, so they are programmed to eat more to compensate for growth and weight gain [17].

In the available literature, there are no current long-term studies that assess the role of the type of delivery, the time of delivery, on obesity in school and preschool children. To prevent the ever-growing global problem of overweight and obesity in childhood, it is necessary to get a deeper understanding of the determinants that must be sought from the time of pregnancy and delivery. The aim of the presented study was to evaluate the influence of type of birth and preterm birth on the long-term risk of childhood overweight and obesity, as well as to assess which of the factors have the greatest impact on the occurrence of excess body weight, taking into account additional maternal factors, such as BMI or gestational weight gain (GWG).

## 2. Materials and Methods

The study was conducted to determine the effect of type of delivery and preterm birth on the future weight of the child and, consequently, on its health. The research was conducted in South-Eastern Poland, in the Podkarpackie Province. First, the authors of the project visited regional educational institutions (in Rzeszów, Kosina, Jarosław, Brzozów, Dębica, and Dynów). During meetings with parents, they presented the main assumptions and goals of the project and distributed consent forms. Parents had the opportunity to ask questions freely and decide on the participation of their child in the study. After obtaining parental consent, the appropriate tests were carried out. 

The study was carried out according to the Declaration of Helsinki and approved by the Ethics Committee of the University of Rzeszów (protocol code No. 18/12/2015 of 2 December 2015).

### 2.1. Participants

The study involved 749 children of pre-school and school age. The average age of the children examined was 9.36 years ± 3.52 years. Children aged 3–18 years were qualified for the study, whose parents gave consent to the child’s participation in the study. The consent of the child was also necessary. 

The 14 institutions participating in the study were selected by random sampling. Parents were informed about the course of the study. The height and weight of each child were measured in the classroom nurse’s room by physiotherapists, and parents completed a questionnaire containing basic information about the child and the family. 

Eligibility criteria included the consent of the parent or legal guardian for their child’s participation in the study, the child’s consent to participate, the child’s age in the range from 3 to 18 years old and a fasting status on examination day. The study group did not include children who did not meet the above eligibility criteria or children with disabilities or lower limb injuries that adversely affect the children’s ability to stand. After receiving consent from the parents, it turned out that the age of the children was within the range of 4–15 years. Furthermore, the children were divided into 3 age groups depending on the level of their education (kindergarten, primary school, and middle school). The first group of children aged 4–6 comprise children attending kindergarten. The age group of 7–11 years is the early stage of school. The age of 12–15 is the school stage.

The sample size was calculated separately for girls and boys due to the fact that the occurrence of excess body weight in children shows a large discrepancy with respect to sex. In the area where the research was carried out, 2,129,138 residents lived. Data show that 51% of them were women and 49% were men, and 14.4% of the female population and 15.8% of the male population were children of preschool and school age [18]. The size of the required sample was calculated, taking into account a confidence level of 95% and the estimated average appearance of the phenomenon of excessive body weight at the level of 20% in girls and 26% in boys [19]. The level of significance was considered to be *p* < 0.05. The minimum sample size for this study was calculated to be 246 girls and 296 boys.

### 2.2. Procedures

#### 2.2.1. Anthropometric Measurements

Body height was measured 3 times (to exclude measurement error) using the Stadiometr Seca 213. The subjects stood barefoot, their backs adjacent to the measuring part of the meter. The device complies with Annex VI of Directive 93/42/EEC regarding medical devices. 

Body weight was assessed using the Tanita BC 420 MA analyzer, which is an analyzer of body weight composition operating on the principle of bioimpedance and is certified by 93/42 EEC (the EU standard for medical devices) [20]. 

The subject stood barefoot on the analyzer, with the upper limbs placed along and slightly away from the torso. 

#### 2.2.2. Preterm Birth and Type of Birth

Information about the type of delivery and potential preterm birth came from the child’s health book and the mother’s pregnancy card. They contained information on the duration of pregnancy and the type of birth (VD or CS). 

After collecting all of the above data, the child’s percentile BMI was calculated, in relation to Polish centile grids and the category of children’s body weight in relation to the classification by Barlow et al. [21,22]:

- Underweight < 5th percentile;
- Normal body weight 5–85 percentile;
- Overweight 85–95 percentile;
- Obesity ≥ 95 percentile.

#### 2.2.3. Statistical Analysis

Data analysis was carried out using Statistica 10.0. Selected methods of descriptive statistics and statistical inference were used. The main numerical characteristics were the number of participants (*n*), percent of participants (%), mean (x), median (Me), and standard deviation(s) of the results. 

Due to the failure to meet the assumptions of the parametric tests (no normality of the distribution in the studied subgroups, analyzed with the Shapiro-Wilk test), the level of statistical significance was calculated using nonparametric tests (chi-square independence tests, the Mann-Whitney test, and the Kruskal-Wallis test). We also used Spearman’s correlation to evaluate the correlation between the BMI of mothers and children. The level of statistical significance was *p* < 0.05.

## 3. Results

### 3.1. Characteristics of the Study Group

**Figure 1:**
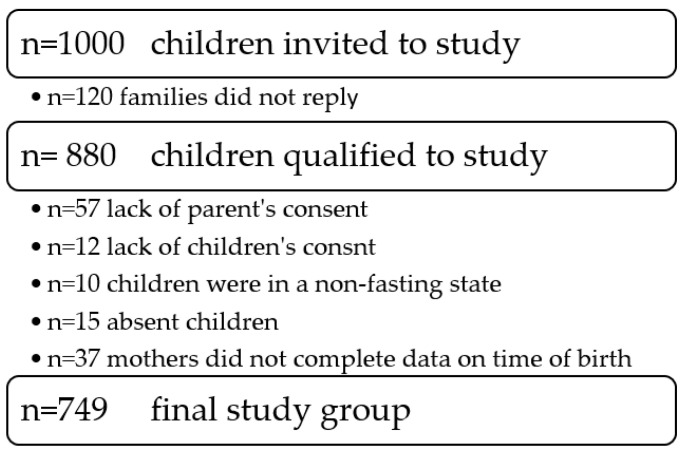
The flow chart of participants.

For purpose of this study, 880 preschool and school children were qualified. Of these, 131 children did not meet the inclusion criteria and finally, 749 children were included in the analysis. In the study group, there were 358 girls and 391 boys, so the criteria for the minimum size of the groups were fulfilled. The detailed flow of the participants is presented in Figure 1.

In the study group, on average, every sixth child was born before the due date (before the end of 37 weeks of gestation) and slightly more than 40% of the children were born by CS. The type of birth and its timeliness were not related to the sex of the newborn (Table 1).

### 3.2. Main Results

In the first step, we checked whether the type of birth influences the BMI percentile value in the examined children, taking into account the sex and age of the subjects. Table 2 summarizes the descriptive statistics values for the BMI centiles in both compared groups and the differences between them were evaluated using the Mann-Whitney test. There were no statistically significant differences.

As in the case of the previous analysis, the results obtained do not indicate a relationship between the type of birth or the incidence of premature birth and the BMI classification of the current body weight of the children (Table 3). Differences were observed in the case of deficiency in body weight (15.4% in the group of premature babies vs. 9.6% in the group of children not burdened with prematurity) and in the group of children with normal body weight (69.9% in the group of premature babies vs. 74.6% in the group of children not burdened with prematurity); however, these differences are not statistically significant.

In individual groups of children, the prevalence of overweight/obesity was compared to type of birth and premature birth. The lack of connection between the type of birth and the category of body weight according to the BMI centile in children is also confirmed by the following analysis. The only exception applies to the group of boys aged 7–11 years, in which a significant difference in the incidence of overweight/obesity in relation to the type of birth appears, to the disadvantage of the CS group (*p* = 0.026) (Table 4).

Finally, a logistic regression analysis was performed to assess the effect of premature and cesarean delivery on the chances of the child becoming overweight and obese. In addition, maternal factors (GWG and BMI) were also taken into account, which may also contribute to the increased body weight in the child. This topic has been discussed in more detail by the authors in another publication [23]. The information about the mother’s BMI concerned the current mother’s BMI—at the time of the child’s examination (school or preschool age, respectively). This information was provided by the mother. Information on GWG was obtained from the maternal pregnancy card and was calculated by the treating physician on the basis of body weight at the beginning of pregnancy and at the end.

The analysis only showed that a 1 kg increase in the weight of a pregnant mother increases the chances of overweight and obesity in school and preschool children by 4%. The other factors turned out to be irrelevant (Table 5).

## 4. Discussion

The current epidemic of childhood obesity is considered a multifactorial etiology condition involving the participation of genetic and environmental factors. Due to the increasing scale of the problem, the causes should be sought from perinatal and postnatal factors [24]. The relationship between CS and the onset of obesity is controversial and is based primarily on the child’s body mass index, which has inherent limitations. There are divergent opinions and scientific evidence regarding the impact of type of birth on the occurrence of overweight and obesity in adulthood. Some studies suggest that the type of birth has a potentially strong effect on long-term health and may affect the body mass index at a later age. 

Darmasseelane et al. conducted a systematic review and meta-analysis of the impact of CS and VD on the BMI of offspring and the appearance of overweight and obesity in adulthood. As a result of a search of available databases, 15 trials with a total population of 163,753 people were identified. The odds ratio for overweight was 1.26 (CI: 1.16–1.38, *p* < 0.001), and for obesity, 1.22 (CI: 1.05–1.42, *p* = 0.01). Similar results were obtained in analyses of subgroups specific for sex [25]. Mamun et al. conducted a study to evaluate the relationship between type of birth and risk of obesity in 21-year-old offspring. In the cohort, 12.1% of the children and adolescents were born through CS, while in our own research, it was up to 41.4% of children and adolescents. At the age of the 21, 21.4% of people who were born naturally and 22.1% of those who were born with CS were overweight. A total of 12.1% of those born naturally and 14.4% of those born by cesarean section were obese, indicating that overweight and obesity were not related to the method of birth [26]. Similarly, Barros et al. evaluated body height, weight, and fat mass at 6, 18 and 30 years and did not confirm a relationship between birth by CS with the fat mass index measured by DXA and the BMI z score in those groups [27].

In our own study, considering the category of current body weight of subjects, no statistically significant differences were found in the occurrence of overweight (CS 11.3% vs. 8.2% VD) and obesity at the level of the entire population (CS 4.8% vs. 7.1% VD), also in the dichotomous division (CS 16.1% vs. 15.3% VD). However, it was observed that the only statistically significant difference was found in the incidence of overweight and obesity in the 7–11-year-old boy group and was 10.7% (CS 19.1% vs. 8.4% VD). Due to the large discrepancy between the available results, further research is needed in this area.

Another important factor that requires analysis is preterm birth. In our own study, 16.4% of children were prematurely born. There were no statistically significant differences in the sexes of the subjects. Considering the category of current body weight of the subjects, there were no statistically significant differences in the prevalence of overweight (premature birth 10.6% vs. 9.3% birth at full term) and obesity in the entire population (preterm birth 4.1% vs. 6.5% delivery at full term), also in a dichotomous division (preterm birth 14.6% vs. 15.8% birth at full term). Although the study did not explicitly confirm the relationship between the factors studied, the topic is worth a more thorough analysis.

Levy et al. conducted an analysis to determine whether premature birth affects children’s health in the long term. The analysis included 225,260 cases, of which 24% (*n* = 54,073) were born prematurely, contrary to our own results, where 16.4% of children and adolescents were born preterm (123 out of 749 examined persons). The incidence of endocrine and metabolic diseases was much more frequent in the group of premature babies (0.51% vs. 0.41%, *p* = 0.003), while overweight and obesity were more frequent in the group of children born prematurely (*p* = 0.002). The differences were more pronounced in children > 5 years of age who had higher rates of type 1 diabetes and obesity. The survival curves showed a higher overall incidence of total endocrine and metabolic morbidity in preterm infants. On this basis, the authors concluded that preterm births are associated with higher percentages of long-term pathology and a higher incidence of endocrine and metabolic diseases compared to births occurring at full term. This association may be due to the lack of full maturity of the hormonal axis in premature babies or alternatively suggest the primary endocrine dysfunction of the fetus as the initial mechanism responsible for spontaneous preterm birth [28]. Hui et al. evaluated the relationship of late prematurity with markers of obesity in adolescence. They also examined whether the acceleration of weight gain of babies is mediated by the analyzed phenomenon. The study included children born prematurely (*n* = 295), compared to children born at full term (*n* = 6874), and compiled with indicators of obesity after 14 years. Children born prematurely had a higher body mass index (BMI) z score (0.21, 95% CI 0.07–0.35), waist circumference z score (0.16; 95% CI 0.03–0.29) and ratio of waist to body height z score (0.27, 95% CI 0.14–0.40) than for children born at full term. Prematurity was associated with higher BMI and waist indicators during adolescence, but only in higher BMI values was it mediated by gain in infant body weight in the first year of life [29].

This study is not free from certain limitations. One of them is the small number of children in particular age subgroups, despite the large total number of examined children. This is probably due to voluntary participation in the study and the fact that the authors did not select children in terms of age. Another limitation is the lack of distinction between planned and emergency cesarean sections performed. Other significant perinatal factors, (e.g., the birth weight of the child or smoking during pregnancy), which, due to the incompleteness of the data, were not included in the presented results, need to be analyzed. Despite the discrepancy in the data in many reports, the study of both prenatal and perinatal factors in the emergence of overweight and obesity in childhood is important today and should continue.

## 5. Conclusions

Preterm delivery does not affect the incidence of overweight and obesity in the study group. Birth by cesarean section significantly increases the percentage of boys with overweight and obesity in early school age and may be associated with higher BMI percentile values among children in this age group. Additionally, it should be noted that there is a large percentage of children born by cesarean section in relation to natural births.

## Figures and Tables

**Table 1:** Preterm birth and the type of delivery depending on the sex of the subjects.

Variables	Sex		
	Girls	Boys	Total
	(*n* = 358)	(*n* = 391)	(*n* = 749)
**Preterm birth (before the end of 37 weeks) (*p*= 0.723)**			
Yes	57 (15.9%) ^a^	66 (16.9%) ^a^	123 (16.4%) ^a^
No	301 (84.1%) ^a^	325 (83.1%) ^a^	626 (83.6%) ^a^
**Type of delivery (*p* = 0.637)**			
Cesarean section	145 (40.5%) ^a^	165 (42.2%) ^a^	310 (41.4%) ^a^
Vaginal birth	213 (59.5%) ^a^	226 (57.8%) ^a^	439 (58.6%) ^a^

^a^—number of subjects and (% of subjects); *n*—number of subjects; *p*—probability test value.

**Table 2:** BMI centile value depending on the type of delivery and preterm birth.

Sex/Age	BMI Centile													
	Type of Delivery						*p*	Preterm Birth (before the End of 37 Weeks)						*p*
	Cesarean Section			Vaginal Birth				Yes			No			
	*n*	Me	IQR	*n*	Me	IQR		*n*	Me	IQR	*n*	Me	IQR	
**Sex**														
girls	145	48.0	53.0	213	46.0	62.0	0.638	57	35.0	59.0	301	49.0	59.0	0.139
boys	165	49.0	50.0	226	44.5	58.0	0.163	66	44.5	61.0	325	48.0	54.0	0.214
**Age**														
4–6 yrs	71	49.0	46.0	107	50.0	56.0	0.875	35	35.0	61.0	143	50.0	49.0	0.133
7–11 yrs	164	48.0	62.0	197	36.0	56.0	0.090	56	36.5	55.0	305	44.0	60.0	0.152
12–15 yrs	75	53.0	44.0	135	50.0	60.0	0.501	32	52.5	54.0	178	50.5	55.0	0.654
**Sex and age**														
girls 4–6 yrs	36	48.0	47.5	52	45.0	59.0	0.424	17	35.0	56.0	71	49.0	53.0	0.195
girls 7–11 yrs	70	49.5	62.0	90	33.0	61.0	0.431	26	26.5	54.0	134	46.0	63.0	0.191
girls 12–15 yrs	39	48.0	53.0	71	60.0	64.0	0.557	14	63.5	69.0	96	53.0	56.5	0.648
boys 4–6 yrs	35	49.0	46.0	55	54.0	65.0	0.351	18	40.0	65.0	72	54.0	49.0	0.392
boys 7–11 yrs	94	48.0	61.0	107	39.0	53.0	0.131	30	45.0	56.0	171	44.0	57.0	0.530
boys 12–15 yrs	36	62.0	42.0	64	41.0	62.5	0.107	18	42.5	64.0	82	47.0	54.0	0.395

IQR—interquartile range; Me—median; *n*—number of subjects; *p*—probability test value.

**Table 3:** Body mass category according to the BMI centile, depending on the type of delivery and preterm birth.

Variables	Underweight	Normal Body Weight	Overweight	Obesity	Total
	(*n* = 79)	(*n* = 553)	(*n* = 71)	(*n* = 46)	(*n* = 749)
**Preterm birth (before the end of 37 weeks) (*p* = 0.181)**					
Yes	19 (15.4%) ^a^	86 (69.9%) ^a^	13 (10.6%) ^a^	5 (4.1%) ^a^	123 (16.4%) ^a^
No	60 (9.6%) ^a^	467 (74.6%) ^a^	58 (9.3%) ^a^	41 (6.5%) ^a^	626 (83.6%) ^a^
**Type of delivery (*p* = 0.164)**					
Cesarean section	27 (8.7%) ^a^	233 (75.2%) ^a^	35 (11.3%) ^a^	15 (4.8%) ^a^	310 (41.4%) ^a^
Vaginal birth	52 (11.8%) ^a^	320 (72.9%) ^a^	36 (8.2%) ^a^	31 (7.1%) ^a^	439 (58.6%) ^a^

^a^—number of subjects and (% of subjects); *n*—number of subjects; *p*—probability test value.

**Table 4:** Incidence of overweight and obesity depending on the type of delivery and preterm birth, with respect to the sex and age of the subjects.

Incidence of Overweight and Obesity with Respect to the Sex and Age	Type of Delivery		*p*	Preterm Birth (before the End of 37 Weeks)		*p*
	Cesarean Section	Vaginal Birth		Yes	No	
**Sex**						
girls	22 (15.2%) ^a^	36 (16.9%) ^a^	0.663	7 (12.3%) ^a^	51 (16.9%) ^a^	0.381
boys	28 (17.0%) ^a^	31 (13.7%) ^a^	0.375	11 (16.7%) ^a^	48 (14.8%) ^a^	0.694
**Age**						
4–6 y	9 (12.7%) ^a^	17 (15.9%) ^a^	0.552	5 (14.3%) ^a^	21 (14.7%) ^a^	0.952
7–11 y	27 (16.5%) ^a^	22 (11.2%) ^a^	0.143	7 (12.5%) ^a^	42 (13.8%) ^a^	0.799
12–15 y	14 (18.7%) ^a^	28 (20.7%) ^a^	0.719	6 (18.8%) ^a^	36 (20.2%) ^a^	0.848
**Sex and age**						
girls 4–6 y	4 (11.1%) ^a^	4 (7.7%) ^a^	0.583	1 (5.9%) ^a^	7 (9.9%) ^a^	0.608
girls 7–11 y	9 (12.9%) ^a^	13 (14.4%) ^a^	0.772	2 (7.7%) ^a^	20 (14.9%) ^a^	0.327
girls 12–15 y	9 (23.1%) ^a^	19 (26.8%) ^a^	0.671	4 (28.6%) ^a^	24 (25.0%) ^a^	0.774
boys 4–6 y	5 (14.3%) ^a^	13 (23.6%) ^a^	0.280	4 (22.2%) ^a^	14 (19.4%) ^a^	0.792
boys 7–11 y	18 (19.1%) ^a^	9 (8.4%) ^a^	0.026	5 (16.7%) ^a^	22 (12.9%) ^a^	0.573
boys 12–15 y	5 (13.9%) ^a^	9 (14.1%) ^a^	0.981	2 (11.1%) ^a^	12 (14.6%) ^a^	0.696

^a^—number of subjects and (% of subjects); *p*—probability test value.

**Table 5:** Logistic regression of the odds of overweight and obesity in children.

Factors	OR (95% CI)	*p*
**All**		
Mother’s BMI	1.020 (0.982–1.060)	0.307
GWG	1.040 (1.011–1.069)	0.006
Preterm birth	1.100 (0.630–1.922)	0.737
Type of delivery	0.957 (0.633–1.448)	0.837
**Boys**		
Mother’s BMI	1.011 (0.965–1.059)	0.649
GWG	1.037 (1.001–1.075)	0.042
Preterm birth	0.910 (0.436–1.899)	0.802
Type of delivery	1.199 (0.676–2.127)	0.535
**Girls**		
Mother’s BMI	1.037 (0.958–1.122)	0.371
GWG	1.049 (0.999–1.101)	0.054
Preterm birth	1.390 (0.581–3.327)	0.459
Type of delivery	0.747 (0.406–1.374)	0.348

## Data Availability

Data available from the authors of this publication.
